# Enhanced Survival of *Plasmodium*-Infected Mosquitoes during Starvation

**DOI:** 10.1371/journal.pone.0040556

**Published:** 2012-07-10

**Authors:** Yang O. Zhao, Sebastian Kurscheid, Yue Zhang, Lei Liu, Lili Zhang, Kelsey Loeliger, Erol Fikrig

**Affiliations:** 1 Section of Infectious Diseases, Department of Internal Medicine, Yale University School of Medicine, New Haven, Connecticut, United States of America; 2 Howard Hughes Medical Institute, Chevy Chase, Maryland, United States of America; Universidade Federal do Rio de Janeiro, Brazil

## Abstract

*Plasmodium* spp. are pathogenic to their vertebrate hosts and also apparently, impose a fitness cost on their insect vectors. We show here, however, that *Plasmodium*-infected mosquitoes survive starvation significantly better than uninfected mosquitoes. This survival advantage during starvation is associated with higher energy resource storage that infected mosquitoes accumulate during period of *Plasmodium* oocyst development. Microarray analysis revealed that the metabolism of sated mosquitoes is altered in the presence of rapidly growing oocysts, including the down-regulation of several enzymes involved in carbohydrate catabolism. In addition, enhanced expression of several insulin-like peptides was observed in *Plasmodium*-infected mosquitoes. Blocking insulin-like signaling pathway resulted in impaired *Plasmodium* development. We conclude that *Plasmodium* infection alters metabolic pathways in mosquitoes, epitomized by enhanced insulin-like signaling – thereby conferring a survival advantage to the insects during periods of starvation. Manipulation of this pathway might provide new strategies to influence the ability of mosquitoes to survive and transmit the protozoa that cause malaria.

## Introduction

Despite recent efforts, malaria remains one of the deadliest infectious diseases in the world. The five *Plasmodium* species (*P. falciparum, P. vivax, P. malariae, P. ovale* and *P. knowlesi*), that cause malaria in humans, are transmitted by female *Anopheles* mosquitoes. For efficient parasite transmission, mosquitoes need to survive long enough to allow for the development of *Plasmodium* oocysts and subsequent invasion of salivary glands by sporozoites. This extrinsic incubation period ranges from 10 to 21 days. As less than 10% of female mosquitoes live longer than 2 weeks in the wild [1], their survival is one of the most vulnerable points in the *Plasmodium* life cycle [2]. For this reason, strategies aimed at decreasing mosquito daily survival rates, including indoor residual insecticide spray and insecticide-treated bed nets, have proven effective in malaria control programs [2]. Other methods aimed at reducing the lifespan of wild mosquitoes have also been proposed and tested [3], [4], [5]. In addition, it has been shown that *Plasmodium* infection sometimes reduces the viability of the mosquito vector, especially at the stage when ookinetes invade the mosquito midgut and elicit an energy demanding immune response by the vector [6], [7], [8]. In general, *Plasmodium* is thought to reduce the overall fitness of its arthropod vector [9].

In nature, mosquitoes are constantly challenged with various stresses, including changes in nutrient availability, temperature, as well as humidity [10]. These environmental conditions interact with the intrinsic genetic factors, such as polymorphisms in mosquito resistance and/or susceptibility alleles, as well as virulent factors of *Plasmodium*, and eventually determine the overall outcome of the infection [11]. For example, low glucose diet supplied to newly emerged mosquitoes has been shown to have negative effects on mosquito survival in addition to *Plasmodium* infection [12]. In the current study, we examined the survival of *Plasmodium*-infected mosquitoes during starvation, and demonstrated that *Plasmodium* influences the carbohydrate metabolism of mosquitoes to provide a survival advantage to the insect vectors.

## Results

### *Plasmodium berghei*-infected *Anopheles* mosquitoes have a better chance to survive under starvation condition

**Figure 1:**
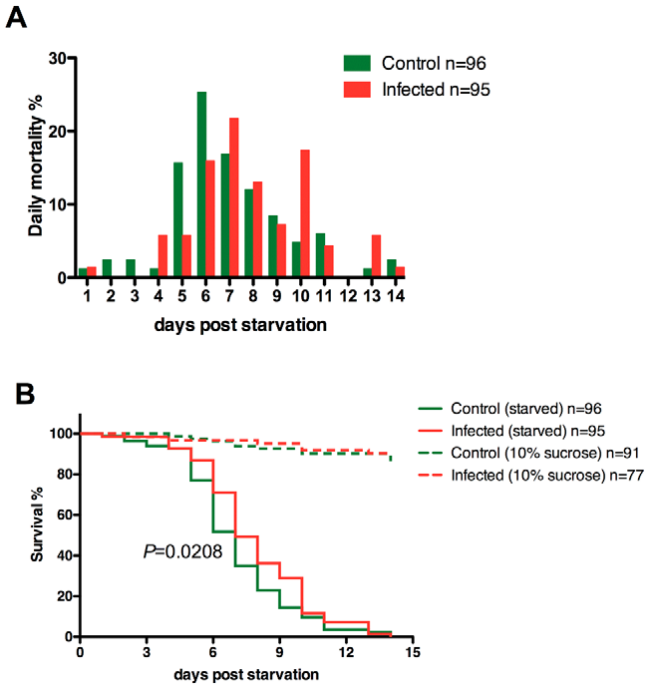
Survival of *Plasmodium berghei*-infected *Anopheles stephensi* at the starvation condition. *A. stephensi* were fed on control or *P. berghei*-infected mice and kept with 10% sucrose for 14 days. The mosquitoes were then deprived of sucrose, or treated with 10% sucrose for comparison. The dead mosquitoes were counted daily. (**A**) Daily mortality of starved *P. berghei*-infected and control A. *stephensi* mosquitoes. (**B**) Cumulative survival curve for indicated groups. The survival curves are significantly different between control and infected mosquitoes under starvation (*P* = 0.02, Gehan-Breslow-Wilcoxon Test). This is one representative data from 3 independent experiments (See Figure S1 for additional data sets).

In our study we infected *A. stephensi* mosquitoes with *P. berghei* and provided them with 10% sucrose for 14 days to allow for the full development of *Plasmodium* oocysts in the mosquito midgut. By the end of this period, released *Plasmodium* sporozoites from mature oocysts started to invade salivary glands and mosquitoes became infectious to mammalian hosts. Mosquitoes were then deprived of sucrose and only provided with water. Daily survival was monitored. Figure 1 shows one representative result from three independent experiments. All mosquitoes died within 2 weeks without food and median survival did not differ between two groups (7 days for both groups). However, while uninfected mosquitoes had a peak in mortality after 6 days of starvation, *P. berghei*-infected mosquitoes showed highest mortality at 7 days after starvation (Figure 1A). The cumulative survival were significantly different between control and infected mosquitoes (Figure 1B, solid lines, *P* = 0.02, Gehan-Breslow-Wilcoxon Test), with the greatest difference in cumulative mortality at 6 days after starvation, when 72% of the infected mosquitoes survived in comparison to 52% of the uninfected insects. The daily mortality for mosquitoes with sufficient food supply during the same period was less than 1%, with no significant difference between two groups (Figure 1B, dashed lines). Similar results were observed from 3 independent experiments (Figure S1, A–B). In addition, this survival advantage during starvation was only observed in mosquitoes with developing oocysts, but not when the mosquitoes were starved immediately after an infectious blood meal (Figure S1, C).

### *Plasmodium*-infected mosquitoes accumulate more glycogens

**Figure 2:**
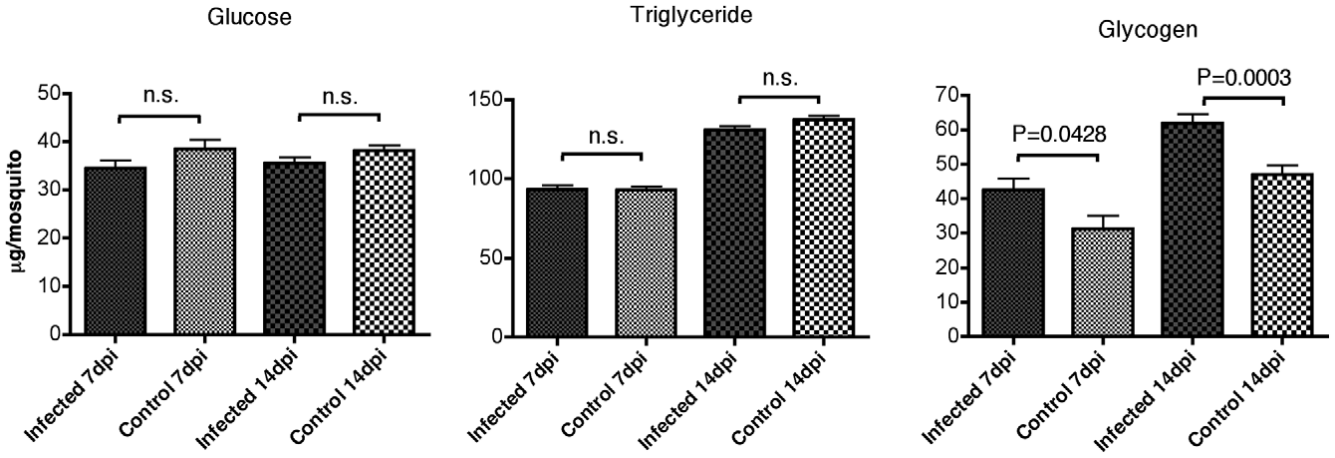
Levels of glucose, triglyceride and glycogen in *P. berghei*-infected and control *A. stephensi* mosquitoes at 7 and 14 dpi. A total of 50–100 mosquitoes were used for each group per assay. Error bars indicate standard deviation and *P* values were analyzed by *t*-test.

One possible explanation for the observed survival advantage of infected mosquitoes only under starvation condition is a difference in energy storage before they are starved. To test this, we examined the levels of energy resources in the mosquitoes provided with sufficient food at 7 and 14 days post infection (dpi). Lipid, glucose and glycogens levels in mosquito whole bodies were determined. As shown in Figure 2, both *P. berghei*-infected and uninfected mosquitoes had comparable levels of glucose and triglyceride contents. However, the glycogen levels in *P. berghei*-infected mosquitoes were significantly higher than those in uninfected animals at both 7 dpi (*P* = 0.04, *t*-test) and 14 dpi (*P* = 0.0003, *t*-test).

### Sucrose uptake is enhanced in *Plasmodium*-infected mosquitoes

**Figure 3:**
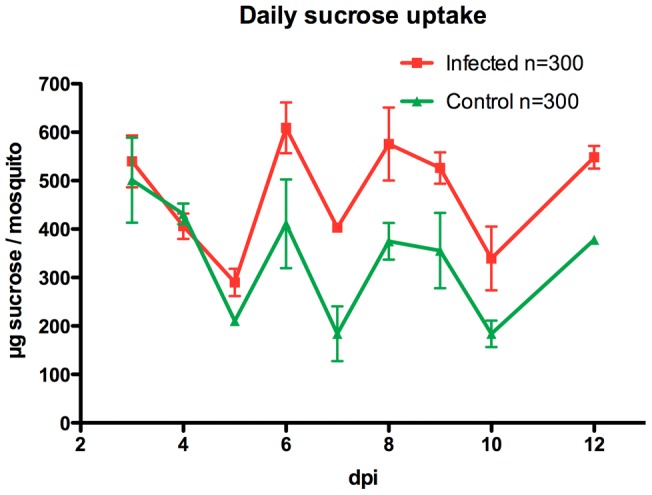
Sucrose uptake from *P. berghei*-infected and control *A. stephensi* mosquitoes after blood meal. Error bars indicate standard deviation from 3 biological replicates from total 300 mosquitoes per group.

Since we observed that *Plasmodium* infection causes higher glycogen accumulation in mosquitoes, we reasoned that this might be due to enhanced food uptake and/or less energy resources consumption. We then directly measured the sucrose uptake of *P. berghei*-infected and uninfected mosquitoes. Figure 3 shows that during periods of blood digestion (up to 4 days after a blood meal at 21°C), both infected and control *A. stephensi* mosquitoes consume similar amount of sucrose solution. However, from 5 dpi, infected mosquitoes consistently ingested up to 2-folds more sucrose per day. Therefore *Plasmodium* infection indeed results in higher amount of food uptake in their mosquito hosts.

### Infection with *P. berghei* causes down-regulation of transcripts associated with carbohydrate catabolism in *A. gambiae* mosquitos during parasite oocyst development

**Figure 4:**
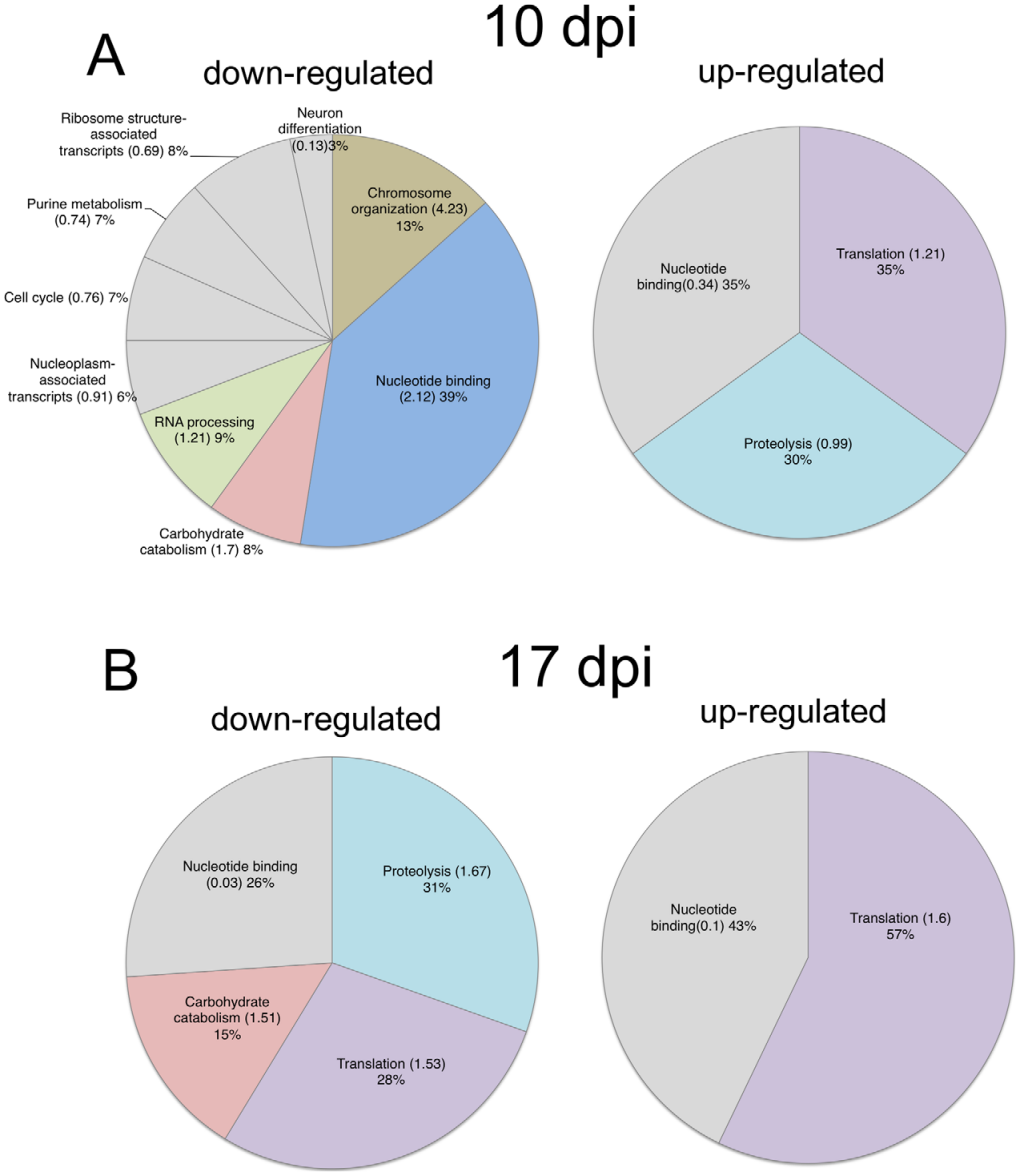
Results of functional annotation clustering of up- and down-regulated *A. gambiae* genes at 10 and 17dpi when compare with uninfected mosquitoes. Clusters with an enrichment score >1 (value in brackets) are colored, clusters with scores <1 are greyed out. (A) At 10 dpi, the down-regulated genes were grouped into 9 discrete clusters, with genes up-regulated in response to *P. berghei* infection grouped into 3 discrete clusters; (B) At 17dpi, the significantly over-represented functions of down-regulated genes are less diverse than 10dpi with carbohydrate catabolism represented at both time points. At this time point up-regulated genes make up 2 clusters.

To further determine the molecular basis of the effect of developing *Plasmodium* oocysts on the mosquito, we performed microarray experiments to compare the gene expression profile of *P. berghei*-infected *A. gambiae* mosquitoes, whose genome sequences are available and microarray studies have been established. Similar to *A. stephensi*, *P. berghei*-infected *A. gambiae* also showed enhanced survival upon starvation (Fig S1, D). We assessed the mosquito gene expression at 10 and 17 dpi, two time points corresponding to oocyst development (10 and 17 dpi) and appearance of sporozoites in the salivary glands (17dpi). A total number of 414 transcripts at 10 dpi and 216 at 17dpi were differentially expressed with at least 1.5 fold change and a false discovery rate (FDR) of 10% or less (Table S1), corresponding to ∼3.0% and ∼1.6% of the mosquito transcriptome, respectively. Among them, 118 (10dpi) and 50 (17dpi) transcripts could be identified to be associated with functional clusters by using DAVID Functional Annotation Clustering algorithm (Huang et al., 2009, Table S2). Figure 4 shows the proportion of either up- or down-regulated transcripts associated with the functional clusters. Overall, we observed that transcripts associated with protein and carbohydrate metabolic functions were over-represented at both time points. In particular, our analysis revealed that several enzymes involved in carbohydrate catabolism were down regulated in infected *A. gambiae* mosquitoes at both 10 and 17 dpi (Table S3). In addition, expression of several genes related to oxidative phosphorylation, lipid degradation (ketone bodies), pentose phosphate pathway as well as TOR-signaling are also down regulated in infected mosquitoes (Table S1). Altogether, our gene expression profile data suggests that the metabolism of mosquitoes is profoundly influenced by the presence of *Plasmodium* infection. Particularly, decreased expression of genes with carbohydrate and lipid catabolic function could lead to decreased energy use and more efficient accumulation of energy storage during a period of sufficient food supply.

### Expression of several insulin-like peptides is up-regulated in *P. berghei*-infected *A. gambiae*

**Figure 5:**
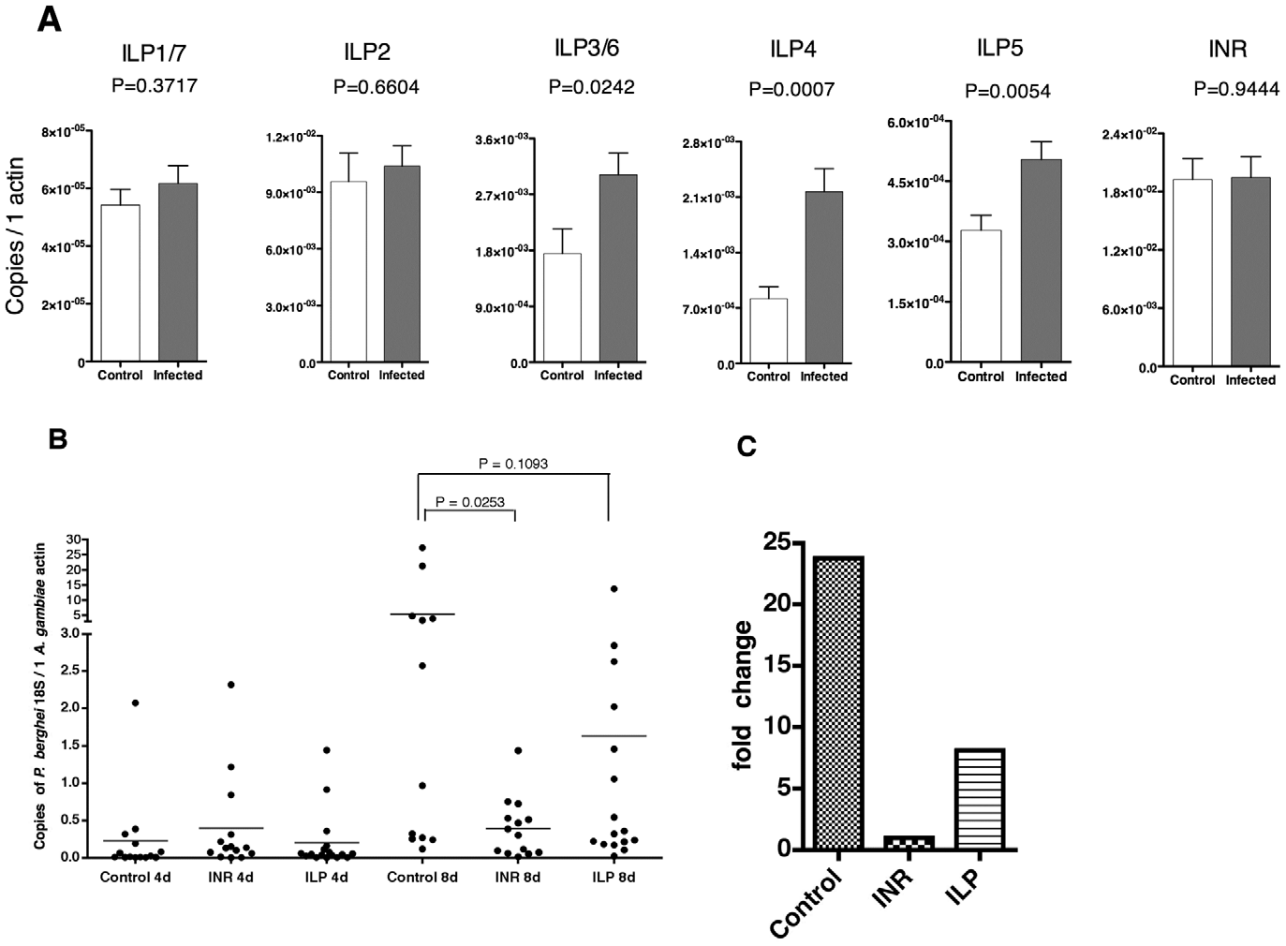
Role of insulin-like signaling in *Anopheles* mosquitoes during *Plasmodium* infection. (**A**) Expression of insulin-like peptides (ILPs) and insulin receptor (INR) gene in *P. berghei*-infected *A. gambiae* at 13 dpi. The expression levels were determined by compared to the expression level of A. *gambiae* actin gene. Error bars indicate standard deviation and *P* values were analyzed by *t*-test. (**B**) Knocking down insulin-like signaling interferes with *Plasmodium* development in the mosquitoes. dsRNA against INR or ILP3/6, 4, 5 (pooled) were injected into *P. berghei*-infected A. *gambiae* at 5 dpi. dsRNA for EGFP were infected as the control. 4 and 8 days after dsRNA injection, the level of *P. berghei* load at the mosquitoes were quantified by qRT-PCR. Each dot represents the *P. berghei* 18S rRNA load in one mosquito normalized to A. gambiae actin gene. Bars indicate mean values and P values were analyzed by Mann-Whitney test. (**C**) Fold changes of *P. berghei* load in the mosquitoes were compared between 4 and 8 days post dsRNA injection.

In all well-studied metazoans, insulin/insulin-like growth factors (IGFs) signaling pathways are the key regulators of metabolism, growth, immunity, as well as longevity [13], [14]. In addition, several reports have shown that microbial infections can modulate the insulin/IGFs signaling in insects. For example, *Mycobacteria marinum*-infected *Drosophila melanogaster* exhibited impaired insulin/IGFs signaling. This resulted in a gradual loss of lipid and glycogen in fly bodies, and infected flies became hyperglycemic [15]. In contrast, flies that were infected with *Wolbachia* had increased insulin/IGFs signaling [16], These observations inspired us to examine the endogenous insulin-like signaling components in *Plasmodium*-infected mosquitoes in more detail. There are 7 insulin-like peptide (ILP) genes in *Anopheles gambiae* genome, four of them being arrayed proximally as duplicate pairs [17]. Quantitative reverse transcription polymerase chain reaction (qRT-PCR) showed that in non-fasting mosquitoes at 13dpi, Ag ILP1, 2 and 7, as well as insulin receptor (INR) have a similar level of expression in infected and uninfected mosquitoes (Figure 5A). However, ILP3/6, 4 and 5 have significantly increased expression in infected animals. Therefore our data suggests that the infection of *Plasmodium* could increase several mosquito insulin-like peptides expression.

### RNAi-induced gene silencing of components of the *A. gambiae* insulin-like pathway interferes with *Plasmodium* development

A role of insulin/IGFs signaling in promoting *Plasmodium* development in their vector hosts was suggested but not formally demonstrated. We therefore employed RNA interference (RNAi) strategy to knock down the expression of insulin-like peptides or insulin receptor in *A. gambiae* infected with *P. berghei*. Double strand (ds) RNA were microinjected into *P. berghei*-infected *A. gambiae* at 5 days post infection, and *Plasmodium* loads were determined at 4 and 8 days following injection (9 and 13 dpi, respectively). No phenotypic change was observed in the groups with individual ILPs knocked down compared to the mock-injected group (data not shown). Considering that the functions of ILPs are probably overlapping and redundant [18], we simultaneously knocked down ILP3/6, 4 and 5 together in one animal (knocking down efficiency shown in Figure S3). As demonstrated in Figure 5B, 4 days after dsRNA injection, no difference in *P. berghei* load in ILP or INR knock down mosquitoes was detected. However, at 8 days after injection, mosquitoes with INR knocked down supported less *P. berghei* development (*P* = 0.025, Mann-Whitney test). While ILP knockdown also reduced *P. berghei* development, the difference was not statistically significant (*P* = 0.109, Mann-Whitney test). In addition, between 4 and 8 days after dsRNA injection, the average *P. berghei* load increased more than 20-fold in the control group, while the level of *P. berghei* remained similar in the INR knock-down group (Figure 5C). Again, ILPs knocking-down had only a moderate effect on *P. berghei* load in the mosquitoes. Taken together we conclude that mosquito insulin-like signaling could boost the late *Plasmodium* development in the mosquito.

## Discussion

Many studies, including lab-based and field studies, have conclusively demonstrated that *Plasmodium* causes pathological changes not only in their vertebrate hosts, but also in insect vectors. This is especially apparent at earlier stages of *Plasmodium* infection in the mosquito, when ookinetes penetrate mosquito midgut epithelium and cause physiological disturbance [6]. Consequently, the mosquito mounts an immune response to control the infection, which is an energetically costly process [9]. In addition, it has been reported that *Plasmodium*-infected mosquitoes have reduced fecundity both in the laboratory and field based studies [19], [20]. Overall, *Plasmodium* infection is thought to be harmful to their mosquito hosts, [7], [9].

It has been also shown that after oocysts established their niche under the basal lamina, the host immune response is gradually diminished and returns to the quiescent level [21]. Thereafter *Plasmodium* oocysts exhibit rapid growth. In support of these earlier observations, we show here by microarray analysis that, during this period, the mosquito metabolism, rather than immunity, is more profoundly affected by the presence of parasites (Figure 4). The growing *Plasmodium* oocysts are apparently an energetic burden on host metabolism, supported by observations including a lower level of free amino acids and sugar in the hemolymph of *P. berghei*-infected mosquitoes [22], [23]. Additional direct evidence is provided by experiments showing that isolated oocysts-containing midguts of *A. aegypti* infected with *P. gallinaceum*, or of *A. stephensi* infected with *P. cynomolgi* utilized up to 8 times more glucose than the uninfected midguts over a 2 h period [6], [24].

Data presented in the current study further demonstrates, however, that in the presence of sufficient food supply, this high energetic burden by itself is not detrimental to the mosquitoes (Figure 1, dashed lines). Instead, we show that the physiology of infected mosquitoes is altered in order to compensate for this energy demand from rapidly growing oocysts. This is directly manifested by enhanced sucrose uptake in laboratory environments. In addition, our microarray data suggests that, the catabolism of energy resources from sated mosquito bodies may be reduced due to down-regulation of some enzymes involved in catabolism of energy storage. Taken together, we hypothesize that *Plasmodium* infection in mosquitoes enhances their food uptake and this enhanced energy input will not only support the development of *Plasmodium* oocysts, but also result in the accumulation of higher energy resource storage in the their own bodies. Consistent with this observation, Rivero and colleagues also found higher glucose and lipid levels in *P. chabaudi*-infected *A. stephensi* mosquitoes at late stage of infection (7 and 14 days)[25]. Higher energy resource accumulation might consequently allow infected individuals better survive periods of starvation.

We consistently observed that under starvation conditions, the maximal survival does not differ between infected and uninfected mosquitoes (Figure 1 and Figure S1). The infected mosquitoes survive better clearly in the early phase during starvation, but later become more susceptible to starvation. Again, this might be due to high-energy drain from *Plasmodium* oocysts. As starvation might minimize the mosquito metabolism and energy flow, it is unlikely able to stop energy utilization by *Plasmodium* oocysts. Further experiments are needed to address the question how mosquito physiology is influenced by dual stresses, namely starvation and *Plasmodium* infection.

In all well studied metazoans, insulin-like peptides signaling plays a central role in the regulation of metabolism, growth, immunity, as well as longevity [13], [14]. In relationship to *Plasmodium* infection in mosquitoes, Beier et al showed early in 1994 that exogenously added human insulin could promote *P. falciparum* survival in mosquitoes [26], while in another published study, expression of constitutively activated Akt, a key downstream molecule in insulin-like signaling in the midgut of *A. stephesi* can simultaneously limit *Plasmodium falciparum* infection and shorten mosquito lifespan [5]. More recently, Marquez and colleagues have provided a detailed study on regulation of *A. stephensi* insulin-like peptides expression within 48 hours of *Plasmodium* infection [27]. These studies however focused on the early stage of *Plasmodium* infection and provided insights into the potential role of insulin-like signaling in mosquito midgut immune response. To our knowledge there is no report so far to investigate the role of mosquito endogenous insulin-like signaling in relationship to late *Plasmodium* development. We reveal now that the expression of several insulin-like peptides is induced in *Plasmodium*-infected mosquitoes at late stage of infection (Figure 5A). This is consistent with the finding that a higher glycogen level is accumulated in infected mosquitoes, as insulin signaling is known to promote energy storage in all known organisms.

Our data further demonstrated that not only enhanced ILPs expression is associated with higher energy resource storage in mosquitoes, but also endogenous insect insulin-like signaling is essential for supporting late *Plasmodium* oocysts development (Figure 5). It is possible that insulin-signaling cascade fulfills this role by regulating synthesis of mosquito nutrient transporter proteins that support *Plasmodium* oocysts growth. It has been shown that the growth of *Plasmodium* oocysts is dependent on the mosquito yolk proteins including vitellogenin and Lipophorin, and knocking down Lipophorin expression results in smaller *Plasmodium* oocysts [28]. Whereas in *A. aegypti* mosquitoes, synthesis of yolk protein vitellgoen and other lipid transporters in mosquito fat body is stimulated by insulin-like peptides and inhibited by dsRNA targeting insulin receptor [29], [30]. Further experiments are needed to test this hypothesis.

We recognize our experiments leave open the possibility that dys-regulated levels of cytokine/growth factors in the blood from *Plasmodium*-infected vertebrate hosts might also play a role in mosquito survival advantage during starvation. Studies by Luckhart and colleagues have indicated that human insulin ingested by the *A. stephensi* could activate mosquito insulin-like signaling cascade and modulate their immune response as well as lifespan [31], [32]. However, since the survival advantage during starvation was not observed when the mosquitoes were starved immediately after an infectious blood meal (Figure S1, C), at least the nutrient content from blood of infected vertebrate hosts is unlikely contributing to this survival advantage during starvation at late stage of infection. It would be of interest to investigate whether *Plasmodium* could manipulate the vertebrate host endocrine system for their benefits in insect hosts.

In summary, we provide evidences that *Plasmodium* infection in mosquitoes dramatically changes the metabolism of their insect hosts to facilitate their own development, and probably also their transmission under certain conditions. Overall the observed enhanced expression of several ILPs, as well as down regulation of some carbohydrate catabolism enzymes, may reflect the adaptation of mosquito metabolism to high-energy requirements resulted from rapid growth of *Plasmodium* oocysts, although the possibility that *Plasmodium* secretes virulence factor(s) to actively manipulate mosquito physiology is not excluded. Since all *Plasmodium* oocysts undergo tremendous expansion during development in mosquito midgut, the revealed phenomenon here might be universal for *Plasmodium* infection in mosquitoes. Further experiments by using more natural *Plasmodium*/mosquito combinations would be necessary to test this prediction. Lastly, it is important to iterate that our finding do not explicitly suggest that *Plasmodium* in general is beneficial for their vector hosts. Consistent with previous reports [19], [20], We also observed that *P. berghei* infected *A. stephensi* and *A. gambiae* mosquitoes produce significantly less eggs. In addition, enhanced food seeking and feeding in nature during early stage of infection might put infected mosquitoes at higher risk of being killed by vertebrate hosts, while energy input from enhanced food uptake is probably essential for *Plasmodium* development. Considering the reduced fecundity, potential higher risk associated with enhanced food seeking, as well as energy cost imposed by mounting an immune response, *Plasmodium*-infected mosquitoes do not gain by being infected. Instead, once infection is established in the mosquitoes, *Plasmodium* could successfully manipulate the vector for its own benefit. Future studies are required to better understand the *Plasmodium*-mosquito interactions, especially in the context of different environmental factors.

## Materials and Methods

### Mosquito rearing

*Anopheles gambiae* (4arr strain, MR4, MRA-121) and *Anopheles stephensi* (ste2, MR4, MRA-128) mosquitoes were raised at 27°C, 80% humidity, under a 12/12 hours light/dark cycle and maintained with 10% sucrose under standard laboratory conditions.

### Ethics statement

Animals were housed and handled under the Guide for the Care and Use of Laboratory Animals of the National Institutes of Health. The animal experimental protocol was approved by the Yale University's Institutional Animal Care & Use Committee (Protocol Permit Number: 2011-07941). All animal infection experiments were performed in a Bio-safety Level 2 animal facility, according to the regulations of Yale University.

### *Plasmodium berghei* infection

*P. berghei* (MR4, ANKA GFPcon 259cl2, MRA-856, or NK65 RedStar, MRA-905) were maintained by serial passage in 4–6 weeks old female Swiss Webster mice (Charles River) from frozen stocks. Mice parasitemia were monitored by light microscopy using air-dried blood smears that were methanol fixed and stained with 10% Giemsa. 3–5 days old mosquitoes were deprived of sucrose for 20 hours and then fed on anesthetized mice (5 mice/cage/200 mosquitoes) 3–5 days after blood inoculation when parasitemia were between 3–6%. Immediately after feeding, all mosquitoes were transferred to and kept at 21°C. 28 hours after feeding, mosquitoes were briefly chilled on ice and fully engorged ones were transferred to new cages at a density of 100–120 mosquitoes per cage (20cm×20cm×20cm, Bioquip, Cat. 1450A). Males and unfed or half-engorged females were discarded. Bottles with 10% sucrose soaked in cotton pad were changed every day. 3–4 days after blood feeding, the mosquitoes were allowed to lay eggs on the wet filter paper. 7–10 days after infection, mosquito midguts were dissected under a fluorescent microscope to confirm the *P. berghei* infection. Cages with infection rate less than 80% were discarded and not further used for experiments. For starvation experiments, mosquitoes were provided with water instead of 10% sucrose from the indicated time points after the blood feeding. Dead mosquitoes were counted and discarded on a daily basis.

### Triglyceride, glycogen, and glucose quantifications

The quantification of energy resources was performed as described [15] with slight modification. Briefly, 5 female A. *stephensi* mosquitoes were homogenized in 0.2 ml Tris-EDTA (TE; 10mM Tris, 1mM EDTA, pH 8) with 0.1% Triton X-100 and 0.01ml of this homogenate was used for each assay. A total of 50–100 mosquitoes were used for each group per assay. Glucose was measured with liquid glucose oxidase reagent (Pointe Laboratories, MI). For measuring glycogen, 1U/ml amyloglucosidase (Sigma-Aldrich, MO) was added into this reagent. The reaction was blanked against the glucose reaction. Triglyceride quantification was measured by using an assay kit from Cayman Chemical Company, MI. All reactions were performed in 96 well plates with 0.2 ml reagent per well.

### Quantification of sucrose uptake

To quantify the amount of sucrose imbibed by infected and uninfected mosquitoes, one cage of each mosquito group was provided with one bottle of 10% sucrose soaked in cotton. Bottle weighs were measured every day. The sucrose bottles were changed every two days. On the second day, sucrose bottles were swapped between infected and control cages to avoid any cage effect. The amount of sucrose uptake were calculated by blanking against sucrose bottles with evaporation only but not accessible to mosquitoes.

### Microarray hybridization, scanning and analysis

Hybridization of samples to the mosquito gene expression microarray from Agilent (G2519F, Design ID 020449, Format 4x44k, 60mer), signal processing, and signal data analysis were performed by Biomedical Genomics Core at The Research Institute at Nationwide Children's Hospital, Columbus, Ohio. For each group per time point, a total of 40 *A. gambiae* mosquitoes from 3 independent cohorts were collected and randomly distributed as four replicates with 10 mosquitoes per group. Every infected mosquito was checked under a fluorescent microscope to confirm *P. berghei* infection before RNA isolation. Total RNA was extracted with the RNeasy Mini kit (Qiagen). RNA concentration was measured on a NanoDrop® ND-1000 UV-Vis Spectrophotometer and RNA integrity was confirmed using an Agilent 2100 Bioanalyzer and Agilent 6000 Series II chips. 50ng of isolated total RNA per sample were reverse transcribed using T7 RNA polymerase and labeled with either Cy3 or Cy5 dyes using the Agilent Low Input Quick Amp labeling procedure, following the manufacturer's instructions (Version 6.5, May 2010). Equal amounts of linearly amplified, Cy3/Cy5-labeled RNA were fragmented by incubating the samples in fragmentation buffer for exactly 30 minutes in a water bath at 60°C; fragmented cRNA were combined in 2x GEx hybridization buffer and the hybridization solution was immediately loaded onto arrays and hybridized in a oven set to 65°C rotating at 10rpm for 17h. Prior to scanning, arrays were washed according go manufacturer's instructions (Agilent Low Input Quick Amp Labeling protocol Version 6.5 May 2010). The microarray data sets have been deposited at the National Center for Biotechnology Information Gene Expresssion Omnibus repository (www.ncbi.nlm.nih.gov/geo) under accession numbers GSE32200.

### Analysis of microarray data for over-representation of gene functional categories

For our analysis, we restricted the expression data by setting minimum thresholds for two parameters: For all time points, we selected transcripts for which an average fold-change of 1.5 (up- or down-regulated) was detected. Additionally, we set a threshold for the False Discovery Rate (FDR) of equal or less than 10%. In total, 535 transcripts met these selection criteria (see Table S1 for a full list of transcripts with annotations). Following the selection based on expression levels and FDR, we analyzed the lists of up- and down-regulated transcripts separately for both time points using the DAVID Functional Annotation Clustering algorithm [33]. The parameters for performing the clustering were determined empirically to eliminate redundancy in the clustering terms which resulted in following values: Similarity Term Overlap –3, Similarity Threshold –0.65, Initial Group Membership –2, Final Group Membership –7, Multiple Linkage Threshold –0.5. The results of the clustering were manually inspected, and the top-level annotation terms describing the functional categories were used to summarize the clustering results in the pie charts presented in Figure 4.

### Evaluation of microarray results by qRT-PCR

We evaluated the differential gene expression results of the microarray, by performing qRT-PCR experiments on a selection of transcripts associated with metabolism, as well as the transcripts with strong up- and down-regulation (Figure S2). Primers were designed using NCBI Primer BLAST and synthesized at the Yale W.M. Keck facility (Table S4). The evaluation confirmed the general trends of the microarray data.

### Quantification of gene expression and *Plasmodium* load

Total RNA was extracted from individual mosquito using the RNeasy Mini Kit (QIAGEN, CA). cDNA was synthesized using the iScript RT-PCR kit (Bio-Rad, CA). Quantitative PCR was performed by using iQ SYBR Green Supermix (Bio-Rad, CA) on a CFX96 real time system (Bio-Rad). PCR involved an initial denaturation at 95°C for 5 min, 45 cycles of 10 sec at 94°C, 10 sec at 60°C, and 10 sec at 72°C. Fluorescence readings were taken at 72°C after each cycle. At the end of each reaction, melting curve (70–95°C) was checked to confirm the identity of the PCR product. Relative expression results were calculated by normalization to A. *gambiae* actin mRNA. *Plasmodium* load was determined by PCR using primers to amplify *P. berghei* 18s rRNA and normalization to *A. gambaie* actin mRNA. For complete list of qRT-PCR primers and accession numbers see Table S4.

### dsRNA synthesis and gene silencing

cDNA from A. *gambiae* 4arr strain was prepared as described before and used as template to amplify DNA encoding a fragment for the targeted genes. Primers were designed by addition of a T7 promoter site (TAATACGACTCACTATAGGGAGA) on 5′ end of both forward and reverse primers. The primer sequences are shown in Table S4. dsRNA were synthesized using the Megascript RNAi kit (Ambion Inc, TX) according to the manufacturer manual. For silencing the target genes, *Plasmodium*-infected mosquitoes were first allowed to lay eggs on the wet filter paper at 3–4 dpi. Mosquitoes were then chilled on ice on 5dpi and ∼200 ng dsRNA in 69nl were injected into mosquito hemocoel through thorax by a Nanojet microinjector (Drummond Scientific, PA). For pooled dsRNA injection, total 200ng dsRNA was used with equal amount of dsRNA for each gene. dsRNA targeting GFP was used as control. After injection, mosquitoes were recovered at 21°C and routinely provided with 10% sucrose throughout their lifespan.

### Statistical analysis

All survival analysis, graphing, and statistics were performed in Prism 4.0 software (GraphPad Software Inc, CA).

## Supporting Information

Figure S1 **Daily mortality and cumulative survival curves of** ***P. berghei*** **-infected** ***A. stephensi*** **(A-C) and** ***A. gambiae*** **(D) when starved at indicated time points (A, B and D, 14 dpi; C, 0dpi) after blood feeding.**
(TIFF)

Figure S2 Evaluation of microarray results by qRT-PCR. Asterisks (*) indicate transcripts that are involved in carbohydrate catabolism (Table S3). AGAP009049 and AGAP005690 are up-regulated transcripts whereas AGAP006191 is a randomly picked transcript that is down-regulated (Table S1).
(TIF)

Figure S3 **Knocking down efficiency from experiments shown in** **Figure 5B** **.** Expression level of indicated genes in *P. berghei*-infected A. *gambiae* from Figure 5B were determined by qRT-PCR compared to the expression level of A. *gambiae* actin gene. Each dot represents the relative expression level of indicated genes in one mosquito normalized to *A. gambiae* actin gene. Bars indicate mean values. Note that there was an enhanced expression of ILP5 at 8 days post injection in animals injected with pooled dsRNA for ILPs, probably due to compensation mechanism.
(TIF)

Table S1 **Differentially expressed transcripts with annotation data.**
(XLSX)

Table S2 **DAVID Functional Annotation Clustering Results.**
(XLSX)

Table S3 **Fold-change values of representative probes targeting transcripts identified to be associated with carbohydrate catabolism.**
(PDF)

Table S4 **Primer sequences.**
(PDF)
