# Supplementary material for: Enhanced Survival of Plasmodium-Infected Mosquitoes during Starvation
Source: PLoS One. 2012 Jul 10;7(7):e40556. doi: 10.1371/journal.pone.0040556 (PMC3393683; doi:10.1371/journal.pone.0040556)
Supplement: Table S3 — Fold-change values of representative probes targeting transcripts identified to be associated with carbohydrate catabolism. (PDF) [file pone.0040556.s006.pdf]

Table S3. Fold-change values of representative probes targeting transcripts identified to be associated with carbohydrate catabolism; *: Transcripts not considered in enrichment analysis due to fold-change and/or FDR not meeting selection criteria.

Gene Name (RefSeq mRNA ID)	INTERPRO Annotation [ID: Name]	KEGG Pathway [ID: Name]	Gene Ontology Biological Process [ID: Name]	Fold change 10 dpi	17 dpi
AgaP_AGAP004197 (XM_313091)	IPR006113: 6-phosphogluconate dehydrogenase, decarboxylating	aga00030: Pentose phosphate pathway, aga00480: Glutathione metabolism	GO:0006007~glucose catabolic process, GO:0006098~pentose-phosphate shunt	-1.649	-1.53
AgaP_AGAP000551 (XM_310532)	IPR001017: Dehydrogenase, E1 component		GO:0006007~glucose catabolic process, GO:0006096~glycolysis	-1.603	-1.52
AgaP_AGAP001903 (XM_321163)	IPR001236: Lactate/malate dehydrogenase	aga00020: Citrate cycle (TCA cycle), aga00620: Pyruvate metabolism, aga00630: Glyoxylate and dicarboxylate metabolism	GO:0006007~glucose catabolic process, GO:0006096~glycolysis	-1.518	-1.521
AgaP_AGAP002914 (XM_311991)	IPR019539: Galactokinase galactose-binding domain	aga00052: Galactose metabolism, aga00520: Amino sugar and nucleotide sugar metabolism,	GO:0005996~monosaccharide metabolic process, GO:0046835~carbohydrate phosphorylation	-1.52	-1.618
AgaP_AGAP003636 (XM_313400)	IPR007828: Protein of unknown function DUF706	aga00053: Ascorbate and aldarate metabolism, aga00562: Inositol phosphate metabolism	GO:0016052~carbohydrate catabolic process, GO:0019310~inositol catabolic process	-1.573	1.074*
AgaP_AGAP006660 (XM_316694)	IPR001804: Isocitrate/isopropylmalate dehydrogenase	aga00020: Citrate cycle (TCA cycle), aga00480: Glutathione metabolism	GO:0006102~isocitrate metabolic process, GO:0055114~oxidation reduction,	-1.742	-1.518
AgaP_AGAP012261 (XM_320278)	IPR001509: NAD-dependent epimerase/dehydratase	aga00052: Galactose metabolism, aga00520: Amino sugar and nucleotide sugar metabolism	GO:0005996~monosaccharide metabolic process, GO:0006012~galactose metabolic process, GO:0019318~hexose metabolic process	-1.561	-1.484
AgaP_AGAP012583 (XM_001230540)	IPR006114: 6-phosphogluconate dehydrogenase, C-terminal,		GO:0006007~glucose catabolic process, GO:0006098~pentose-phosphate shunt	-1.7	-1.644
AgaP_AGAP007827 (XM_317672)	IPR000941: Enolase	aga00010: Glycolysis / Gluconeogenesis, aga03018: RNA degradation	GO:0006007~glucose catabolic process, GO:0006096~glycolysis	-1.648	-1.604
AgaP_AGAP004451 (XM_313741)	IPR001937: Galactose-1-phosphate uridyl transferase, class I	aga00052: Galactose metabolism, aga00520: Amino sugar and nucleotide sugar metabolism	GO:0005996~monosaccharide metabolic process, GO:0006012~galactose metabolic process	-1.298*	-1.758
