# Supplementary material for: Enhanced Survival of Plasmodium-Infected Mosquitoes during Starvation
Source: PLoS One. 2012 Jul 10;7(7):e40556. doi: 10.1371/journal.pone.0040556 (PMC3393683; doi:10.1371/journal.pone.0040556)
Supplement: Table S4 — Primer sequences. (PDF) [file pone.0040556.s007.pdf]

Gene	ID	Forward	Reverse	product size(bp)
Primers for qRT-PCR				
P. berghei 18s rRNA	160646	CCGACTAAGTGTTGGATGAAAA	TACTCGCCCCAGAACCCAAAGA	119
A. gambiae actin	AGAP000651	GAAGGCTAACCGCGAGAAGATG	CGCCGGAGTCCAGCACGATA	134
A. gambiae ILP1/7	AGAP010603/AGAP010605	TACTGCTCGTTCTGCTCTCC	AGCAACATTCGGCCACCAC	355
A. gambiae ILP2	AGAP010600	AAAGCCACGCTACCTCTAC	CACCTCCACCTTTACTCGCTG	184
A. gambiae ILP3/6	AGAP010602/AGAP010604	GGTAAAGGTACTGTCCTTCCTG	TGTTGTCTTCCGCGTTCATC	128
A. gambiae ILP4	AGAP010601	AGCTGCCGAAGATACTTGCC	CCATTCTCACTAAAGTCCCAGTCC	142
A. gambiae ILP5	AGAP003927	CTGATACGCCATCTGTACTGG	TACGCTTTTCCACCATCCCG	100
A. gambiae INR	AGAP012424	CCAACCTTACCAGGGACTGA	GCATCGGGTAACAACATACG	180
A. gambiae Enolase	AGAP007827	GCCGCGTCCGAGTTCCACAA	GTCAACCAGGCGCTCGGGTC	80
A. gambiae 6-phosphogluconate dehydrogenase	AGAP004197	GCGGACCGCAAGCAGTTCCT	CGAATGATGCAACCGCCGCG	167
AGAP003636	AGAP003636	CGGACAACCGACTGCACCAGC	ACACACAAGCACGCTTTTTCACCC	107
AGAP006191	AGAP006191	ACCGACTGAATATGACGAGCAAACC	GATGGGTGCACAGGTCCGGG	117
AGAP005090	AGAP005090	ACTCCTCGGGAGCCAAAGCCT	CGATCCATTCGGCGCTGGCA	125
AGAP009049	AGAP009049	GGACGCTCTGGCCCGTACACA	ATCGCCGCTAACCGCATACGG	123
Primers for dsRNA synthesis: a T7 promotor (taatacgactcactatagggaga) was added to 5' of both forward and reverse primers				
A. gambiae ILP3/6	AGAP010602/AGAP010604	CAACTTCGCCAAGCCGTCGGA	TACCGCACGGCCGCAAACAA	257
A. gambiae ILP4	AGAP010601	GGACTGGGACTTTAGTGAGAATGG	AGTGGGCACCACTCCTGGTG	253
A. gambiae ILP5	AGAP003927	TCGAGACGCAGCGGCGATGG	TCGCCGGTACTGGTTCAAGCG	258
A. gambiae INR	AGAP012424	CACACCCACCACGTTGTGCG	TCCGAGCGGCTAAGTCGCGA	253
GFP		GCGACGTAAACGGCCACAAGTT	CGCGGGTCTTGTAGTTGCCGTC	270
